# The College of Nursing, Limited

**Published:** 1917-11-10

**Authors:** 


### The College of Nursing, Ltd.

CERTIFICATED NURSES were accepted for membership of the College by the Council at its last meeting who had received their training at one of the following training-schools:

LONDON AND PROVINCIAL TRAINING-SCHOOLS.

London Hospital, E.; Prince of Wales' General Hospital, Tottenham, N.; St. Bartholomew's Hospital, London; Hospital of St. John and St. Elizabeth, London; St. Thomas's Hospital, London; University College Hospital, London; West London Hospital, Hammersmith, W.; St. Pancras Infirmary North, Highgate, N.; Southwark Infirmary, London; West Ham Infirmary, Leytonstone; Ashton-under-Lyne District Infirmary; Bury Infirmary, Lancs; Cheltenham General Hospital; Royal Infirmary, Halifax; Royal Infirmary, Huddersfield; Warneford, Leamington, and South Warwickshire General Hospital; General Infirmary, Leeds; Royal Infirmary, Manchester; Royal Hospital, Salford, Manchester; Royal Infirmary, Sunderland; Royal Sussex County Hospital, Brighton; Ashton-under-Lyne Poor-Law Institution; Dudley Road Infirmary, Birmingham; Greenwich Infirmary; Kingston-on-Thames, Surrey; Brownlow Hill Poor-Law Institution, Liverpool; Steyning Poor-Law Institution, Shoreham; Tonbridge Poor-Law Institution, Tunbridge Wells; Wolverhampton Poor-Law Institution; the affiliated certificate of the Seamen's Hospital, Greenwich, and the Royal Waterloo Hospital for Children and Women.

SCOTTISH TRAINING-SCHOOLS.

Royal Infirmary, Edinburgh; Royal Infirmary, Glasgow; Western Infirmary, Glasgow; Victoria Infirmary, Glasgow; Royal Infirmary, Aberdeen; Royal Infirmary, Dundee; Royal Alexandra Infirmary, Paisley; Northern Infirmary, Inverness; Kilmarnock Infirmary; Deaconess Hospital, Edinburgh; Eastern District Hospital, Glasgow; Western District Hospital, Glasgow; Stobhill Hospital, Glasgow; Merryflatts Hospital, Govan.

IRISH TRAINING-SCHOOLS.

Royal City of Dublin Hospital; Richmond, Whitworth, and Hardwicke Hospitals, Dublin; Drumcondra Hospitals, Dublin; Mater Misericordiæ Hospital, Dublin; Meath Hospital, Dublin; Royal Victoria Hospital, Belfast; Mater Infirmorum Hospital, Belfast; Union Infirmary, Belfast; County Antrim Infirmary.

For Nursing Affairs in Ireland, see p. 120.

